# NitroGene: privacy-preserving collaborative genomic analysis using AWS nitro enclaves

**DOI:** 10.1093/bioadv/vbag191

**Published:** 2026-07-13

**Authors:** Zakariya Ali, Arif Harmanci

**Affiliations:** Department of Health Data Science and Artificial Intelligence, D. Bradley McWilliams School of Biomedical Informatics, The University of Texas Health Science Center Houston, Houston, TX 77030, United States; Department of Health Data Science and Artificial Intelligence, D. Bradley McWilliams School of Biomedical Informatics, The University of Texas Health Science Center Houston, Houston, TX 77030, United States

## Abstract

**Motivation:**

Protecting participant privacy is a major challenge in large-scale biomedical collaborations. We present NitroGene, an open-source framework for privacy-preserving collaborative genomic analysis using AWS Nitro Enclaves. NitroGene uses an architecture consisting of a client application, proxy server, and hardware-isolated enclave, secured through end-to-end 256-bit encryption and cryptographic attestation via the Nitro Security Module. The framework enables multiple institutions to jointly analyse pooled genomic data without exposing raw data to other participants or the server operator. Each participant encrypts and uploads data files, which are decrypted, merged, and analysed within the enclave before encrypted results are returned to each participant. NitroGene is application-agnostic and supports existing analysis tools without modification by encapsulating them in Docker images.

**Results:**

We demonstrate that NitroGene produces results that are concordant with centrally pooled analyses across three representative genomic applications: collaborative principal component analysis (PCA) on 2712 subjects, cross-cohort kinship estimation involving 32 604 pairwise comparisons, and collaborative genome-wide association studies (GWAS) on 1 million SNPs. Results show that NitroGene enables privacy-preserving collaborative genomic analysis while maintaining compatibility with existing analysis workflows.

**Availability and implementation:**

NitroGene is publicly available on Github (https://github.com/zakkaz1/NitroGene) including the documentation to test the three analysis pipelines and for building new pipelines.

## 1 Introduction

Modern genomic studies require large sample sizes for statistical power, and recent research has been accelerated by the massive increase of individual genomic datasets (The 1000 Genomes Project Consortium 2015, Brittain *et al.* 2017). Population genetics, genome-wide association studies (Witte 2010), and ancestry studies can benefit from diverse, multi-population cohorts (Bentley *et al.* 2017, Bergström *et al.* 2020). However, there are numerous challenges for achieving this goal. For example, individual research institutions often have limited sample size or population diversity. Additionally, large scale computational analyses such as GWAS and genotype imputation can be resource intensive. Lastly, smaller institutions may lack computation infrastructure for large-scale analyses.

Potential solutions include conducting collaborative analyses that pool datasets from multiple institutions or otherwise outsourcing computation to third-party servers with specialized infrastructure. However, genomic data represent highly sensitive personal information and privacy regulations (i.e. GDPR, HIPAA) (Shabani and Marelli 2019) and data sharing agreements can limit raw data sharing and the number of potential collaborators (Aziz *et al.* 2019, Bonomi *et al*. 2020, Cho *et al.* 2024). Similarly, participants can be reluctant to upload unencrypted genomic data to external servers or share with other institutions, due to risks such as data breaches or data misuse (Wang *et al.* 2017, Bonomi *et al*. 2020, Almosti and Rahman 2025).

Although approaches such as homomorphic encryption (Gentry 2009, Mott *et al.* 2020, Kim *et al.* 2021, Li *et al.* 2023, Cho *et al.* 2025) and multiparty computation (Orlandi 2011, Cho *et al.* 2018) can provide theoretical security guarantees, these methods are challenging to develop, maintain, and deploy due to resource requirements (Supplementary Information Section S8). Alternatively, trusted execution environments provide privacy protections by separating memory and CPU at the hardware level, Intel SGX, have been utilized (Kockan *et al.* 2020, Nilsson *et al.* 2020, Froelicher *et al.* 2021, Cho *et al.* 2024, Rauscher *et al.* 2025). For example, the PRINCESS framework used Intel SGX to securely run distributed rare-disease genomic analyses (Chen *et al.* 2017). Similarly, Dokmai *et al*. (2021) used Intel SGX for secure genotype imputation within an enclave while achieving accuracy comparable to standard imputation tools. While these approaches are promising, the documentation and codebases are scattered and may be challenging to modify for implementing new pipelines by general users (Naehrig *et al.* 2011).

In this application note, we aim to build a collaborative tool, NitroGene, that uses AWS Nitro Enclaves, which are hardware-isolated compute environments within Amazon EC2 instances providing dedicated vCPUs and memory with no persistent storage, no external network access, and cryptographic attestation signed by the Nitro Hypervisor (Amazon Web Services n.d). By utilizing these enclaves, NitroGene relies on the hardware isolation (both at the physical instance level on AWS cloud and at the CPU/memory level) so that sensitive data remains protected throughout computation and processing. This framework supports both individual outsourced analysis and multi-party collaboration. We also present end-to-end implementations of cross-cohort principal component analysis, kinship estimation, and genome-wide association study (GWAS), which demonstrates evidence for the feasibility of hardware-isolated genomic computation at scale. NitroGene makes two primary contributions. First, it introduces a multi-party collaboration protocol including session management, slot-based participant coordination, per-participant encryption, which are capabilities not provided by the AWS Nitro Enclave infrastructure. Second, it bridges hardware-based confidential computing, such as vsock communication, Nitro Security Module interaction, and attestation parsing, with increased ease of use through simple command-line invocations.

## 2 Methods

### 2.1 AWS nitro enclaves

Hardware isolation works via AWS Nitro Enclaves, which provide dedicated vCPUs and memory with no external network access. Furthermore, cryptographic attestation is handled by the AWS Nitro Hypervisor, which signs an attestation document which can be used to prove the identity and integrity of the enclave. The attestation document includes platform configuration registers (PCRs), which are unique hashes generated from specific enclave measurements including the enclave image file, kernel data, parent instance, and running application. These PCRs are utilized to verify that all components and code within the enclave match those expected based on the provided enclave image file. Expected PCR values are published by the enclave image administrator, an independent entity responsible for managing and deploying the analysis pipeline. Furthermore, the attestation document includes a nonce provided by the client to ensure freshness and prevent replay attacks (National Institute of Standards and Technology n.d). Lastly, the attestation document includes the enclave’s public key which is used for data encryption and secure communication between client and enclave. The end-to-end encryption utilizes AES-256-GCM for VCF data and ECDH P-384 for key exchange.

### 2.2 NitroGene architecture

NitroGene consists of three primary components: client application, proxy server, and enclave application. The client application handles data encryption, data upload, attestation verification, and result decryption. The proxy server, which acts as an intermediary who does not receive plaintext individual level data, manages collaboration, packages and routes encrypted data to the enclave application via VSOCK protocol. Finally, the enclave application performs decryption of submitted data, analysis in hardware isolation, and encryption of results, and the application operates entirely in memory with no disk I/O. The framework is mainly built using Python 3, involving cryptography libraries, and Bash scripts for launching and managing Nitro Enclave. NitroGene also includes documentation and scripts for using PLINK 1.9 (Purcell *et al.* 2007), GCTA (Yang *et al.* 2011), and KING (Manichaikul *et al.* 2010) within new applications (Supplementary Information Section S1, S2).

### 2.3 Multi-party collaboration protocol

In collaborative tasks, one of the sites initializes the collaboration by submitting a collaboration request via the client application to the proxy server. This collaboration request includes a user-generated nonce and the total number of participants. The proxy server transmits a unique collaboration ID and attestation document to the leader. After the client application programmatically verifies the attestation document, the application uploads the encrypted data. The leader is responsible for securely sharing the credentials with the collaborators. This phase of the multi-party collaboration involves dynamic participation by collaborators, who only require the collaboration ID to engage in joint analysis (Supplementary Information Section S3, S4).

After receiving a collaboration ID and slot number, each participant independently generates their own nonce and symmetric key, performs attestation with the enclave, and performs their own verification of their connection to the proxy server and enclave, and submits their data (Client-to-Enclave Protocol below). The proxy server stores per-participant encrypted results, and each participant independently fetches encrypted results using their assigned slot number. Note that proxy server observes only encrypted data traffic and relays data to and from enclave.

### 2.4 Client-to-enclave protocol

After receiving the collaboration ID and slot number, each client communicates only with the proxy server using a secure channel. Our workflow involves individual key management in which each participant generates and stores symmetric keys locally. The workflow involves five phases.

- Client requests an attestation document and verifies the enclave identity via PCR values. This request is appended with a nonce, which aims at ensuring the attestation document is a fresh copy. The attestation document includes a public key generated by the enclave, which is used to set up a secure channel.
- Client generates and encrypts a symmetric key using the enclave’s public key. Client also encrypts its sensitive data with the symmetric key, which are both sent to the proxy server.
- Proxy server packages the data and forwards it to the enclave via VSOCK protocol, and the enclave decrypts and conducts the analysis.
- Enclave application encrypts the results with the participant’s symmetric key and returns the data to the client via the proxy server.

**Figure 1:**
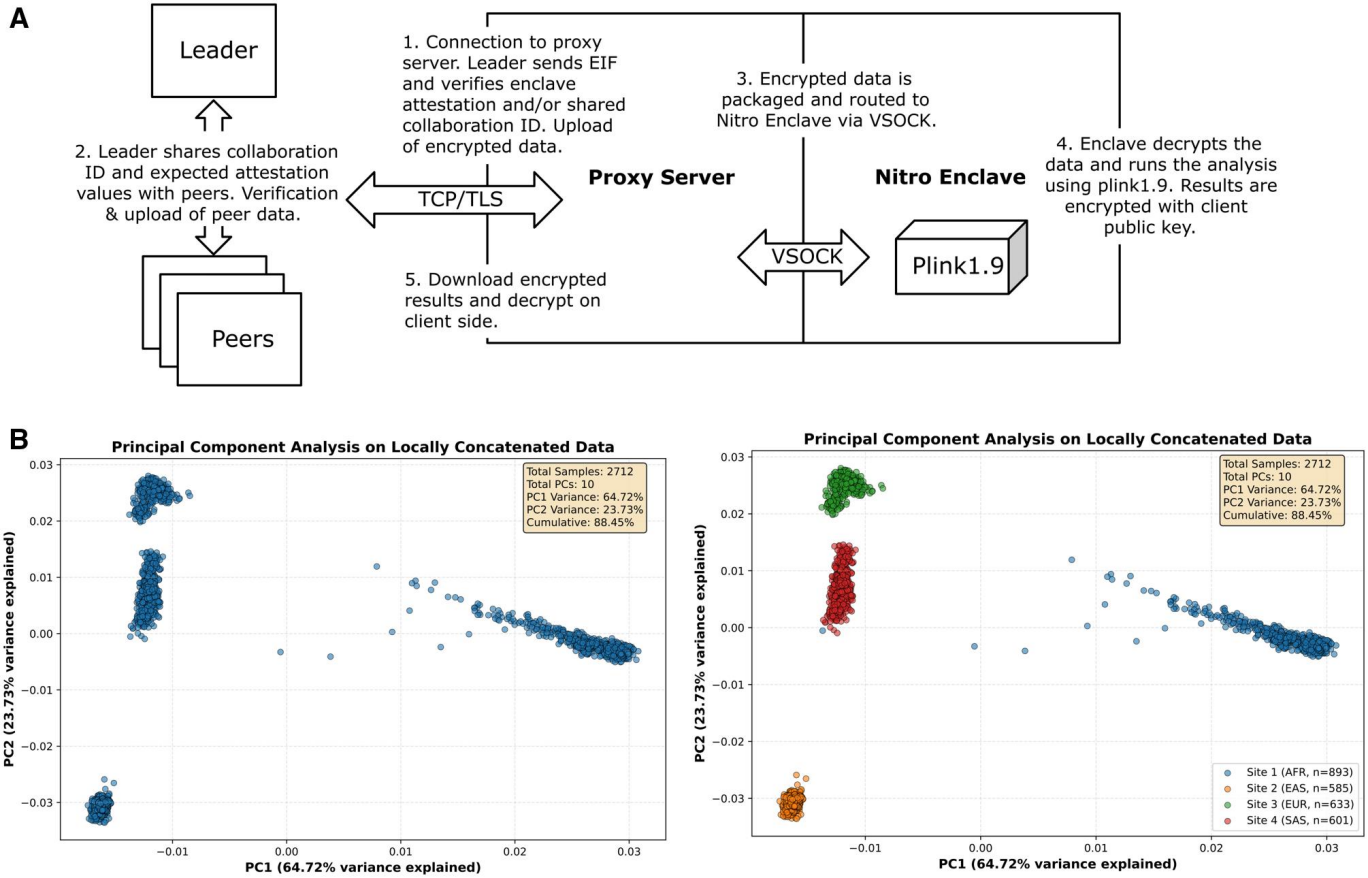
Overview of NitroGene architecture, workflow, and case study result. (A) Architecture and workflow: NitroGene consists of a client application, proxy server, and enclave application. The collaboration between clients consists of a leader and their peers. All clients have access to the expected PCR values associated with the trusted enclave image file. Client-to-Enclave protocols resumes: (i) The leader initiates a collaboration and obtains the collaboration ID from the proxy server. The client encrypts genomic data, verifies enclave attestation, and submits analysis requests. (ii) The leader communicates to the collaborators out of scope and transmits the collaboration ID. The collaborators can then communicate with the server, verify the enclave connection, encrypt, and upload data securely. (iii) The proxy server packages and routes encrypted data to the enclave. (iv) The enclave decrypts the data, performs analysis using PLINK 1.9 entirely in memory, encrypts the results with each participant’s symmetric key. (v) Results are returned. (B) Collaborative PCA: Two PCA plots are shown for comparison. The first illustrates the PCA structure that would result if genotype data from the four client sites were locally merged and analysed in a traditional environment. The second shows the PCA computed using NitroGene, in which the same datasets were encrypted, jointly analysed inside the enclave, and returned to each participant without exposing raw data. Four 1000Genomes superpopulations (AFR, EAS, EUR, SAS) were contributed by separate client sites and subsequently were pooled inside the enclave (2712 samples). Principal component analysis revealed clear separation of the four populations, with PC1 explaining 64.7% of the variance and PC2 explaining 23.7%.

The overall workflow of NitroGene’s protocols is shown in Fig. 1A (Supplementary Information Section S2, S3).

## 3 Results

### 3.1 Collaborative PCA

We started from the 1000 Genomes Project [2] and selected samples from four superpopulations, namely African (AFR), East Asian (EAS), European (EUR), and South Asian (SAS), totaling 2712 samples, and subsampled the variants to 20 000 common SNPs using BCFTools (Danecek *et al.* 2021). The VCF files are distributed in the repository for reproduction of the analysis, which sets up a realistic scenario in which multiple institutions have genomic data for diverse samples. Using NitroGene, we executed the collaborative PCA protocol (collaboration setup, followed by the client-to-enclave communications) using PLINK-based validated docker images across four client sites with each site contributing a distinct super-population on c6a.2xlarge instances. In total, 2712 samples were pooled and analysed inside the enclave. The projection on first two components revealed an exact match of the four superpopulations (PC1 and PC2 explaining 64.7%, 23.7% of the variance, respectively), demonstrating that NitroGene’s protocol can accurately reproduce population-level genetic patterns (Fig. 1B).

### 3.2 Cross-cohort kinship and GWAS

We have additionally implemented two other protocols in addition to the collaborative PCA. These include the cross-cohort kinship estimation using KING (Manichaikul *et al.* 2010), and a collaboartive GWAS analysis using PLINK. In both applications, NitroGene generated results show high concordance to the pooled cohort results (Fig. S1, S2, Supplementary Information Section S5, S6). Time requirements of analysis are highly similar to the plaintext pooled analysis (44 vs 54 seconds for GWAS, Supplementary Section S7). The overhead of our protocol stems from encryption/decryption, VSOCK transfer of encrypted data from proxy sever to the enclave. These estimates do not include the encrypted data uploads and downloads rely on the network status.

### 3.3 Threat models

NitroGene assumes an honest-but-curious adversary model for the clients and server: The clients and proxy server do not actively or maliciously try to break protocols but they may observe data leakage. Under this model, the proxy can observe metadata (encrypted file sizes, upload timestamps, collaboration IDs, participant counts) but cannot access plaintext sensitive data or symmetric encryption keys (Table S1). Co-participants receive only their own individually encrypted results and never receive copies of sensitive data. A malicious proxy server can invite new collaborators to the workflow as they have access to the collaboration ID. This should, assuming AWS root of trust, not leak any sensitive data to the unauthorized collaborators. Furthermore, the security assumptions of the framework rely on the CPU/memory isolation imposed by a valid hypervisor. Any systemwide attack on AWS infrastructure (e.g. supply chain attacks) may taint the hypervisors and leak information from Nitro enclaves. While this would require a successful infrastructure-wide attack on AWS and is highly unlikely, we do acknowledge the possibility. Assuming the security of Nitro system and hardware root of trust, external adversaries attempting to eavesdrop on communications or perform man-in-the-middle attacks should not be able to obtain encryption keys or access plaintext genomic datasets. Any metadata sent across entities and proxy server must utilize explicit encryption such as TLS in large scale and long-term deployments (Supplementary Information Section S7–9).

### 3.4 Comparison to existing approaches

NitroGene connects cloud-native hardware-isolation-based security with collaborative analysis protocols that can be used to build privacy-enhancing pipelines (Supplementary Information Section S8). In its current design, NitroGene protects against honest-but-curious entities and well-designed analysis images should not leak sensitive information across clients, the main server, or an external party listening to the secure channel since all sensitive data is transmitted in encrypted form. However, metadata such as number of collaborating clients, and collaboration IDs are observed at the proxy server, who may accidentally or intentionally leak it. As such, NitroGene’s vulnerabilities should be well understood while it is being used in collaborations.

NitroGene offers an alternative route to complement other approaches to improve practicality of theoretically secure approaches to close any metadata leakages and to harden hardware-level protections. Among these, homomorphic encryption allows computation on ciphertext but incurs orders-of-magnitude overhead and typically requires algorithm-specific reimplementation (Harmanci *et al.* 2021, Kim *et al.* 2021, Cho *et al.* 2024). Secure multi-party computation provides strong theoretical guarantees but introduces substantial communication overhead and requires all parties online throughout computation (Cho *et al.* 2018). By allowing complex algorithms to run natively (e.g. PLINK) inside the enclave with near-native performance, NitroGene can be used to take over data protection at the compute heavy tasks while other parts of pipelines can be protected by HE. This compatibility extends across analyses: The enclave image is built from a standard Docker container, and any command-line tool that operates on sensitive data can be included. Future development is required for extending collaborative tasks via library-agnostic integration of the HE/MPC (Dowlin *et al.* 2017, Albrecht *et al.* 2018, Benaissa *et al.* 2021, Mouchet *et al.* 2021) primitives.

## Supplementary Material

vbag191_Supplementary_Data

## Data Availability

The data underlying this article are available in Github at https://github.com/zakkaz1/NitroGene/.
