# Supplementary material for: NitroGene: privacy-preserving collaborative genomic analysis using AWS nitro enclaves
Source: Bioinform Adv. 2026 Jul 13;6(1):vbag191. doi: 10.1093/bioadv/vbag191 (PMC13479745; doi:10.1093/bioadv/vbag191)
Supplement: Supplementary file 1 [file vbag191_supplementary_data.docx]

**Supplemental Information for the manuscript**

**“NitroGene: Privacy-Preserving Collaborative Genomic Analysis Using AWS Nitro Enclaves”**

Zakariya Ali ^1^, Arif Harmanci ^1^

^1^ Department of Health Data Science and Artificial Intelligence, D. Bradley McWilliams School of Biomedical Informatics, The University of Texas Health Science Center Houston, TX, 77030, USA.

# S1. Cryptographic Protocol

NitroGene uses a layered encryption architecture so that plaintext genomic data exists only inside the hardware-isolated Nitro Enclave and the proxy operates as an intermediary who does not receive individual level plaintext sensitive data.

## S1.1. Key Generation

At boot, the enclave generates an EC P-384 (secp384r1) key pair in memory (enclave_core.py); the private key is never serialized to disk, and the public key is exported in DER SubjectPublicKeyInfo format for inclusion in the attestation document. P-384 provides 192-bit security, exceeding the 128-bit NIST minimum for long-term protection. Each client generates a fresh 256-bit AES key per session using secrets.token_bytes(32), saved locally as symmetric_key_slot_N.bin. Symmetric keys are independent across participants.

## S1.2. Client-Side Encryption

Each VCF is encrypted with AES-256-GCM under the participant’s symmetric key, using a fresh random 96-bit IV. Output bytes are concatenated as IV (12 B) ‖ Tag (16 B) ‖ Ciphertext. The symmetric key itself is transported to the enclave under an Elliptic Curve Diffie–Hellman exchange: the client generates an ephemeral EC P-384 key pair, performs ECDH against the enclave’s attested public key, derives a 256-bit key-encryption key via HKDF-SHA256 (info=b'symmetric key encryption'), and AES-256-GCM-encrypts the symmetric key under the KEK. The transmitted blob is Ephemeral Public Key DER ‖ IV ‖ Tag ‖ Encrypted Symmetric Key. Fresh ephemeral key pairs per session provide forward secrecy at the key-exchange level.

## S1.3. Enclave-Side Decryption and Result Encryption

Inside the enclave the process is reversed: ECDH with the client’s ephemeral public key reproduces the KEK, which unwraps the symmetric key; the symmetric key then decrypts the VCF. All decrypted data lives only in enclave memory and is cleared on completion. After analysis, results are serialized to JSON and re-encrypted separately per participant under that participant’s symmetric key. Only the corresponding client can decrypt their own results; the proxy handles ciphertext only.

# S2. Attestation and Key Exchange

## S2.1. NSM Interaction

The enclave communicates with the AWS Nitro Security Module via /dev/nsm using CBOR-encoded ioctl messages. The attestation request carries an optional user_data, a client-provided 32-byte nonce, and the enclave’s DER-encoded public key. The NSM returns a COSE_Sign1 structure (RFC 8152) whose payload contains module identification, SHA-384 PCR values (PCR0–PCR15, 48 bytes each), a certificate chain, the supplied nonce, and the enclave’s public key, all signed by the Nitro Hypervisor. PCR0 measures the enclave image, PCR1 the kernel and boot parameters, and PCR2 the enclave application.

## S2.2. Client-Side Verification

On receipt of an attestation document, the client (verify_attestation_document): (1) parses the COSE_Sign1 structure as a four-element CBOR array; (2) verifies the COSE_Sign1 signature against the embedded AWS Nitro CA certificate chain, anchored at the AWS Nitro root CA; (3) verifies that the embedded nonce matches the one the client generated for this session; (4) validates PCR0, PCR1, and PCR2 against the expected measurements in expected_pcrs.json; and (5) extracts the enclave’s EC P-384 public key for the subsequent key wrap. Any failure aborts the session before any encrypted data is transmitted.

## S2.3. Anti-Replay and Anti-Impersonation

Replay is prevented by the per-session 32-byte nonce. Enclave impersonation requires forging the Nitro Hypervisor signature, which is infeasible without the hardware signing key. Participant impersonation within a collaboration is mitigated by client_id binding (S3): each slot is bound to the client that claimed it, and submissions from any other client_id are rejected.

# S3. Multi-Party Collaboration Protocol

AWS Nitro Enclaves provide hardware isolation but no built-in multi-party session management. NitroGene adds this layer on top.

## S3.1. Session Initialization and Joining

The leader sends a create_collaboration message specifying the number of expected participants and the analysis type. The proxy generates a 256-bit collaboration ID via secrets.token_urlsafe(32) (a 43-character URL-safe string), allocates the requested slots, auto-assigns the leader to slot 1, and returns the collaboration ID and assigned slot. The collaboration ID serves as both the session identifier and the shared access secret; the leader distributes it to intended collaborators through an out-of-band channel. Each collaborator then presents the collaboration ID to the proxy, which validates it, assigns the next available slot, and binds the participant’s client_id to the slot.

## S3.2. VCF Submission

Each participant independently verifies an attestation document (S2), generates a fresh AES-256 symmetric key, encrypts the VCF under it, wraps the symmetric key under the enclave’s attested public key (S1.2), and submits both to the proxy. The proxy validates that the submitting client_id owns the target slot, stores the encrypted payloads, and tracks submission status.

## S3.3. Enclave Processing

When all N participants have submitted, the proxy connects to the enclave over vsock (port 5000) and sends the full batch in a single process_collaboration message. Inside the enclave, in hardware-isolated memory, each symmetric key is unwrapped via ECDH, each VCF is decrypted and written to tmpfs, the VCFs are converted to PLINK binary format and merged (plink --vcf --make-bed, plink --merge-list), PCA is computed (plink --pca 10), and the eigenvalues, eigenvectors, and variance-explained statistics are serialized to JSON. Results are then encrypted separately per participant and returned to the proxy. The tmpfs work directory is deleted and decrypted data is cleared from memory on completion.

## S3.4. Result Retrieval

Each participant polls the proxy with fetch_collaboration_results and receives one of collaboration_not_ready, collaboration_failed, or collaboration_results (containing the encrypted result for the requesting slot). The client decrypts locally with its saved symmetric key.

# S4. Communication Protocol

## S4.1. Message Framing

Control messages use length-prefixed JSON framing (a 4-byte big-endian length followed by a JSON body). Bulk payloads (encrypted VCFs and encrypted results) use a binary frame protocol: a length-prefixed JSON header followed by one or more length-prefixed raw ciphertext payloads. This avoids hex-in-JSON overhead and allows streaming reception.

## S4.2. Vsock Communication

Proxy–enclave communication uses the vsock interface, the only permitted channel for Nitro Enclaves. The enclave listens on AF_VSOCK (CID=VMADDR_CID_ANY, port 5000). The proxy connects using the enclave’s CID (from nitro-cli describe-enclaves). The vsock channel is exclusive to the parent EC2 instance.

## S4.3. Message Types

| **Message Type** | **Direction** | **Purpose** |
| --- | --- | --- |
| request_attestation | Client → Proxy | Request enclave attestation with client nonce |
| attestation_response | Proxy → Client | Return attestation document (hex-encoded) |
| create_collaboration | Client → Proxy | Initialize a new collaboration session |
| collaboration_created | Proxy → Client | Return collaboration ID and assigned slot |
| join_collaboration | Client → Proxy | Join with collaboration ID |
| collaboration_joined | Proxy → Client | Confirm join and return assigned slot |
| submit_collaboration_vcf | Client → Proxy | Submit encrypted VCF and encrypted key |
| process_collaboration | Proxy → Enclave | Send all encrypted VCFs for batch analysis |
| collaboration_results | Enclave → Proxy | Return per-participant encrypted results (binary frame) |
| fetch_collaboration_results | Client → Proxy | Request results for a specific slot |
| nonce_challenge | Proxy → Enclave | Forward client nonce for attestation generation |

## S4.4. Metadata Exposure

Under the honest-but-curious threat model the proxy can observe collaboration IDs, participant counts, encrypted file sizes (which correlate with sample and variant counts), timestamps, client IP addresses, and enclave processing time. It cannot observe VCF contents, AES symmetric keys, results, or the enclave’s private key. Padding and VPN tunneling are recommended where metadata leakage matters.

# S5. Deployment and Reproducibility

## S5.1. Enclave Build

scripts/build_enclave.sh validates prerequisites; stages the enclave core, the variant’s enclave_app.py, and required binaries; builds an amazonlinux:2-based Docker image; converts it to an EIF with nitro-cli build-enclave; and records PCR0/PCR1/PCR2 to expected_pcrs.json. Any change to enclave code or dependencies produces new PCRs, by design.

## S5.2. Server Deployment

On a Nitro-enabled EC2 instance (m5.xlarge or larger): install aws-nitro-enclaves-cli and Docker; allocate at least 2 vCPUs and 2 GB to enclaves in /etc/nitro_enclaves/allocator.yaml; place PLINK 1.9 in bin/; build the EIF with sudo scripts/build_enclave.sh pca; launch the enclave with sudo nitro-cli run-enclave --eif-path nitrogene-pca.eif --cpu-count 2 --memory 2048 --enclave-name nitrogene-pca; and start the proxy with python3 secure_proxy_server.py --host 0.0.0.0 --port 8443 --enclave-name nitrogene-pca.

## S5.3. Client Setup and Case Study

Clients need Python 3.9+ with the conda environment activated. No AWS account or enclave access is required on the client side. To reproduce the PCA case study, clone the repository (https://github.com/zakkaz1/NitroGene) and deploy the proxy as above. The repository ships pre-prepared VCFs for four 1000 Genomes superpopulations (AFR, EAS, EUR, SAS), subsampled to 20,000 random SNPs. One client runs create-collab --num-collaborators 4; the remaining three run join-collab with the shared collaboration ID. Each participant then fetches and plots their results with fetch-results and plot-results. Expected output: PC1 ≈ 64.7% and PC2 ≈ 23.7% variance explained, with clean separation of the four superpopulations.

## S5.4. Extending NitroGene

Adding a new analysis requires creating enclave_apps/<variant>/ with a Dockerfile and an enclave_app.py that implements run_analysis(participants, work_dir) and calls run_enclave from enclave_core.py; adding any required binaries to the Dockerfile and to the variant prerequisite list in scripts/build_enclave.sh; and rebuilding with sudo scripts/build_enclave.sh <variant>. The client and proxy are agnostic to the analysis. Existing variants (PCA, KING, GWAS) serve as templates.

# S6. GWAS and Kinship Concordance with Plaintext Joint Analysis

To verify that encryption and enclave isolation introduce no numerical artifacts, we compared the output of the NitroGene enclave pipeline against a naive plaintext pipeline in which the three cohort genotype files are pooled on a single machine and the same tool (KING or PLINK2) is run on the merged dataset. Both pipelines apply identical preprocessing (position-deduplication, FID rewriting to slot labels, allele-coding harmonization) so the inputs to the analysis step are byte-identical. All runs use 1000 Genomes Phase 3 chr22 (2,504 samples), split three ways with seed 42.

## S6.1. KING Cross-Site Kinship

We ran king --related --degree 3 on the merged three-cohort dataset and compared the kinship coefficient (φ) reported for every between-family (cross-cohort) sample pair. Across 32,604 cross-site pairs, the NitroGene and plaintext pipelines produced byte-identical kinship coefficients (Pearson r = 1.000000, max |Δφ| = 0, RMSD = 0). KING is deterministic given identical input bytes; the enclave’s isolation does not perturb the computation.

**Figure S1.** KING cross-site kinship concordance. Each point is one cross-cohort sample pair (n = 32,604). The red line is y = x. Pearson r = 1.000000; max |Δφ| = 0; RMSD = 0.

## S6.2. GWAS Linear Association

We ran plink2 --glm under the linear model with 10 joint PCs as covariates and a simulated quantitative trait (GCTA --simu-qt, 100 causal SNPs, h² = 0.5). Across 1,051,671 variants tested, the NitroGene and plaintext pipelines agreed to Pearson r = 1.000000 on -log₁₀(P) values (max |Δlog₁₀P| = 2.16 × 10⁻⁴, RMSD = 3.72 × 10⁻⁵). Both pipelines reported the same 1,983 genome-wide significant hits (P < 5 × 10⁻⁸). Residual numerical differences are below the precision at which PLINK2 reports -log₁₀(P) and reflect the limits of floating-point output formatting rather than a divergent computation.

**Figure S2.** GWAS P-value concordance. Each point is one variant (n = 1,051,671). The red line is y = x. Pearson r = 1.000000; max |Δlog₁₀P| = 2.16 × 10⁻⁴; RMSD = 3.72 × 10⁻⁵. Both pipelines identify the same 1,983 genome-wide significant hits at P < 5 × 10⁻⁸.

## S6.3. Interpretation

The concordance results confirm that the NitroGene framework is faithful to the underlying analysis tools: the encryption layer, the vsock transport, and the enclave isolation contribute no numerical perturbation to KING kinship estimation, and only floating-point output-precision noise to PLINK2 GWAS P-values. The framework therefore offers privacy without sacrificing analytic equivalence to a conventional pooled analysis.

# S7. Limitations

We summarize known limitations of the current implementation, distinguishing architectural constraints from practical engineering gaps that could be addressed in future work.

## S7.1. Output Privacy

All participants receive the full eigenvector matrix, including sample identifiers and PC coordinates for every sample across all participants. PC coordinates are linear projections of genotype vectors and could in principle support inference of population membership or, with external reference panels, partial genotype reconstruction. Mitigations include returning only eigenvalues, returning only each participant's own eigenvectors, or applying differential privacy noise; this trade-off is shared by any collaborative PCA that returns full eigenvectors to all parties. More broadly, NitroGene provides computational privacy on inputs but does not bound any individual's contribution to the output, and calibrating DP noise to preserve analytical utility for population genetics remains a candidate for future work. The proxy itself observes collaboration IDs, participant counts, encrypted file sizes, timestamps, IP addresses, and enclave processing time; file size correlates with sample and variant count. Padding, traffic shaping, and VPN tunneling are recommended for high-sensitivity deployments but are not built into the framework.

## S7.2. Transport and Session Security

VCF payloads are protected end-to-end by AES-256-GCM, but JSON control messages travel between the client and the proxy over raw TCP rather than TLS; we recommend an SSH tunnel or VPN for production deployments, and native TLS support is planned.

## S7.3. Hardware Trust Assumptions and Cloud Provider Coupling

The security model relies on the AWS Nitro Hypervisor and the hardware root of trust: that the hypervisor isolates enclave memory from the host, that the attestation signing key cannot be extracted, and that the NSM faithfully reports enclave measurements. A firmware-level compromise or supply-chain attack would invalidate confidentiality. This is fundamental to any TEE-based approach and is shared by Intel SGX, AMD SEV, and ARM TrustZone. Side-channel attacks (timing, cache, power) are a general concern; Nitro Enclaves mitigate cache timing channels via dedicated CPUs and memory regions, and the coarse granularity of timing information visible to the proxy limits practical exploitability. NitroGene depends on AWS-specific infrastructure: Nitro-enabled EC2, the AWS NSM, nitro-cli, and the Nitro Enclaves allocator. The cryptographic and collaboration protocols are portable in principle, but the attestation mechanism and enclave lifecycle are AWS-specific. Provider-agnostic TEE abstraction is a candidate direction; differences in attestation protocols across providers make this non-trivial.

## S7.4. Tested Scale

PCA protocol in the main text evaluates 2,712 samples across 20,000 SNPs from four participants. The applications of NitroGene presented in Section S6 extend the framework to 1000 Genomes chr22 (~1M variants) for KING and GWAS, but systematic benchmarking across larger cohorts, denser variant sets, and more participants is needed to characterize the framework’s scalability envelope at production scale.

For the GWAS validation run on the same c6a.2xlarge instance (enclave allocated 4 vCPUs and 8 GB of memory, matching the resources available to a plaintext baseline on the parent), the enclave-side wall-clock time for the analysis was 53.8 s, compared to 44.0 s for an equivalent unencrypted analysis executed directly on the host. The difference includes the cost of per-participant key unwrap, result encryption, and vsock result transport in addition to any overhead intrinsic to the enclave isolation. Client–proxy data transport (encrypted upload and result download) added 109.7 s and 6.57 s respectively and scales with input/output size rather than the cryptographic protocol.

# S8. Comparison to Cryptographic Approaches

NitroGene’s underlying architecture is based on hardware isolation and attestation, which are inherently different from cryptographic approaches such as Homomorphic Encryption and multiparty computation. It is therefore important to compare these approaches. The key distinctions are: (1) Homomorphic encryption (HE)[1–4] allows direct computation on ciphertext. To accomplish this, every function must be first re-formulated to HE-native addition and multiplication operations. As HE incurs major penalty on multiplication operations and accumulates noise on every operation, HE functions are implemented by careful optimizations to minimize multiplicative circuit depth. For example, any non-linear operation must be approximated, e.g. polynomial expansions. While most HE libraries have made major progress in the last decade, these HE implementations still incur orders-of-magnitude extra computational overhead. Small adjustments in algorithms may require HE tools to be re-implemented, which makes these approaches very application and version specific. Furthermore, HE does not natively support collaborative operations. For this purpose, multiparty or multikey HE libraries must be used, which further increases storage and computational requirements. (2) Secure multi-party computation (MPC)[5] is another cryptographic approach to perform secure data analysis by creating data shares, analyzing each share in physically separate nodes and aggregating results securely across nodes. MPC provides strong theoretical guarantees but introduces substantial communication overhead and requires all parties to remain online throughout computation. Similar to HE, MPC requires the algorithms to be fully re-designed, approximated, and optimized using MPC-based primitives. (3) TEE-based approaches like NitroGene allow existing unmodified analysis tools (e.g., PLINK[6]) to run on plaintext data inside the enclave, offering near-native performance with no algorithm redesign. (4) The cloud-based confidential computing platforms do not impose explicit memory limitations and allow physical separation of hardware as they run on cloud instances, which makes them practically most attractive for deployment purposes. However, they rely strongly on root of trust and reliable hardware isolation by Nitro system. The trade-off is reliance on trust on the providers (e.g., AWS) and hardening assumptions.

# S9. Adversarial Capabilities and Mitigations

NitroGene’s collaborative protocol does not protect against all threat actors. To systematically document possible routes of attacks, we include an inclusive list of curious/malicious entities that can perform various attacks on the collaborative protocol implemented in NitroGene in Supplementary Table S1.

| **Adversary** | **Capabilities** | **Can access genomic data?** | **Mitigations** | **Remaining limitations** |
| --- | --- | --- | --- | --- |
| **Honest-but-curious proxy server operator** | Observes all traffic routed through proxy; controls parent EC2 instance | No plaintext VCFs, keys, or results | AES-256-GCM encryption, ECDH key wrapping, Nitro Enclave isolation, attestation verification | Can observe metadata (participant counts, file sizes, timestamps, collaboration IDs) |
| **External passive network observer** | Observes network traffic | No plaintext genomic data | End-to-end encrypted payloads; attestation-based key exchange | Metadata exposure; control messages currently not TLS-protected |
| **Active network attacker** | Intercepts, drops, replays, or modifies packets | No direct access to genomic data | Nonce-based replay protection; attestation verification; AES-GCM integrity protection | Denial-of-service possible; TLS is needed to protect metadata |
| **Malicious participant** | Participates in collaboration and receives outputs | Only sees outputs intentionally returned by analysis | Per-participant encryption; slot binding; client-ID validation | May infer information from released outputs (e.g. PCA coordinates) |
| **Compromised host operating system** | Full control of parent EC2 instance | Cannot directly access enclave memory | Hardware-enforced enclave isolation; attestation | Can observe metadata and disrupt service |
| **Malicious or compromised enclave image** | Runs attacker-controlled code inside enclave | Yes | PCR verification against trusted measurements; attestation validation | Security depends on trusted distribution of enclave image and PCR measurements. |
| **Compromised AWS Nitro trust chain / firmware-level attacker** | Breaks attestation or isolation guarantees | Potentially yes | None within NitroGene; trust assumption of Nitro hardware root of trust | Out of scope; fundamental limitation of TEE-based systems |
| **Side-channel attacker** | Exploits timing/cache/power side channels | Potentially partial leakage | Dedicated CPUs and memory regions reduce some channels | Cannot be fully eliminated in AWS infrastructure. |

**Supplemental Table S1:** Threat actors, potential attacks, and mitigations.

# REFERENCES

1.	Dowlin N, Gilad-Bachrach R, Laine K, Lauter K, Naehrig M, Wernsing J. Manual for using homomorphic encryption for bioinformatics. Proc IEEE Inst Electr Electron Eng. 2017;105: 1-16;

2.	Gentry C. A FULLY HOMOMORPHIC ENCRYPTION SCHEME. PhD Thesis. 2009; 1–209.

3.	Harmanci AO, Kim M, Wang S, Li W, Song Y, Lauter KE, et al. Open Imputation Server provides secure Imputation services with provable genomic privacy. bioRxiv. 2021; 2021.09.30.462262.

4.	Kim M, Harmanci AO, Bossuat J-P, Carpov S, Cheon JH, Chillotti I, et al. Ultrafast homomorphic encryption models enable secure outsourcing of genotype imputation. Cell Systems. 2021;12: 1108-1120.e4.

5.	Orlandi C. Is multiparty computation any good in practice? ICASSP, IEEE International Conference on Acoustics, Speech and Signal Processing - Proceedings. 2011. doi:10.1109/ICASSP.2011.5947691

6.	Purcell S, Neale B, Todd-Brown K, Thomas L, Ferreira MAR, Bender D, et al. PLINK: A tool set for whole-genome association and population-based linkage analyses. Am J Hum Genet. 2007;81: 559–575.
